# Review of Prediction Models to Estimate Activity-Related Energy Expenditure in Children and Adolescents

**DOI:** 10.1155/2010/489304

**Published:** 2010-06-29

**Authors:** Suzanne M. de Graauw, Janke F. de Groot, Marco van Brussel, Marjolein F. Streur, Tim Takken

**Affiliations:** ^1^Department of Physiotherapy Sciences, School of Clinical Health Sciences, Utrecht University, NL-3584CX Utrecht, The Netherlands; ^2^De kleine Plantage, Stek Youth Care, NL-3065RG Rotterdam, The Netherlands; ^3^Child Development & Exercise Centre, Wilhelmina Children's Hospital, University Medical Centre, NL-3508AB Utrecht, The Netherlands; ^4^University of Applied Sciences, NL-3584CJ Utrecht, The Netherlands; ^5^Meerweide, Nursery home, de Stromen Opmaat Groep, NL-3078RC Rotterdam, The Netherlands

## Abstract

*Purpose*. To critically review the validity of accelerometry-based prediction models to estimate activity energy expenditure (AEE) in children and adolescents. *Methods*. The CINAHL, EMBASE, PsycINFO, and PubMed/MEDLINE databases were searched. Inclusion criteria were development or validation of an accelerometer-based prediction model for the estimation of AEE in healthy children or adolescents (6–18 years), criterion measure: indirect calorimetry, or doubly labelled water, and language: Dutch, English or German. *Results*. Nine studies were included. Median methodological quality was 5.5 ± 2.0 IR (out of a maximum 10 points). Prediction models combining heart rate and counts explained 86–91% of the variance in measured AEE. A prediction model based on a triaxial accelerometer explained 90%. Models derived during free-living explained up to 45%. *Conclusions*. Accelerometry-based prediction models may provide an accurate estimate of AEE in children on a group level. Best results are retrieved when the model combines accelerometer counts with heart rate or when a triaxial accelerometer is used. Future development of AEE prediction models applicable to free-living scenarios is needed.

## 1. Introduction

Physical activity is defined as any bodily movement produced by skeletal muscles that results in energy expenditure (EE) [1]. Research has shown that there is a positive relationship between physical activity and health-related fitness [2, 3]. Health-related fitness refers to those components of fitness that benefit from a physically active lifestyle and relate to health [4]. The relationships between physical activity, health-related fitness, and health status are described by Bouchard et al. [4]. 

Valid and reliable instruments are necessary, when examining the dose-response relationship between physical activity and health-related fitness [5].

Estimation of physical activity and energy expenditure in children is difficult since children show physical activities of varying intensity and short of duration [6]. Subjective techniques are less preferable in children because of their complex movement behavior and their ability to accurately recall intensity, frequency, and duration of their activities [5].

Direct observation is considered a gold standard for the assessment of physical activity. Gold standard methods to assess activity related energy expenditure (AEE) are doubly labelled water and indirect calorimetry [5, 7]. These methods are mainly used for calibration and validation of objective and subjective measurements in laboratory and field settings. Due to their costs and invasiveness are these methods less suitable for population-based studies [5, 7]. There is a need for accurate, objective, and cost-effective methods to asses AEE in children in free-living situations.

Accelerometry is objective as well as cost-effective and less invasive. Accelerometers have evolved from simple mechanical instruments to electronically three-dimensional instruments to assess physical activity and energy expenditure. An accelerometer estimates accelerations produced by movement of a body segment or limb parts [8]. Acceleration is the change in velocity over time of the body part as it moves. Electronic transducers and microprocessors convert recorded accelerations into digital signals, which are the “counts”. In research, the counts can be used as an estimation of physical activity. Prediction models convert these counts in EE or AEE [8]. AEE can be derived from EE by subtracting resting energy expenditure (REE). Researchers and clinicians prefer predicting AEE instead of gross EE because REE can vary with age, maturation, body mass and level of physical activity [9].

In literature different prediction models are described to assess AEE in children and adolescents. The aim of this study is to review the validity and generalizability of accelerometry based prediction models to estimate AEE in children and adolescents.

## 2. Method

### 2.1. Literature Search

Electronic bibliographic databases CINAHL, EMBASE, PsycINFO, and PubMed/MEDLINE were searched till April 2009. The following MeSH terms and text words were used: child*, adolescent*, youth, physical activity, energy expenditure, accelerometer, accelerometers, accelerometry, uniaxial accelerometer, biaxial accelerometer, triaxial accelerometer, motion sensor, motion sensors, activity monitor, activity monitors, validity, validation, equation, prediction model, calibration, and reproducibility of results.

Studies (written as full reports) were included in this review if their main purpose was to develop and/or validate an accelerometry based prediction model for the estimation of AEE in healthy children and/or adolescents (6–18 years). The AEE predicted by the model, had to be compared with a criterion measure of AEE as doubly labelled water or indirect calorimetry. Studies written in Dutch, English, and German were included. Studies concerning pedometers were excluded.

One researcher (SdG) performed the search strategy. The first selection regarding relevance, based on title and abstract, was performed by two independent researchers (SdG and MS). Furthermore, the included articles were judged on full-text by these two independent researchers. References of the included articles were screened for additional eligible studies.

### 2.2. Data Extraction

To evaluate and compare the studies, data were extracted. Two reviewers independently extracted the data (SdG and MS). Disagreements between the two reviewers regarding a study's eligibility were resolved by discussion until consensus was reached or, when necessary, a third person (JdG) acted as adjudicator.

The data extraction was based on items that have an impact on the range and generalizability of a prediction model according to Puyau et al. [10] and Trost et al. [11]. These items were age range, setting, type of activities, and localisation of the accelerometer. Additionally accelerometer type, criterion measure, prediction models, and conclusions were extracted.

### 2.3. Evaluation

An existing checklist [12] was modified to evaluate and compare the studies regarding methodological issues (see Appendix A). Checklist items included study design, validity, reliability, and feasibility. Maximum possible score was 10, high score reflected a better methodological quality. Two reviewers independently scored all included studies on the checklist (SdG and MS). A third reviewer (JdG) was consulted when the reviewers did not reach consensus.

## 3. Results

### 3.1. Search Result

**Figure 1:**
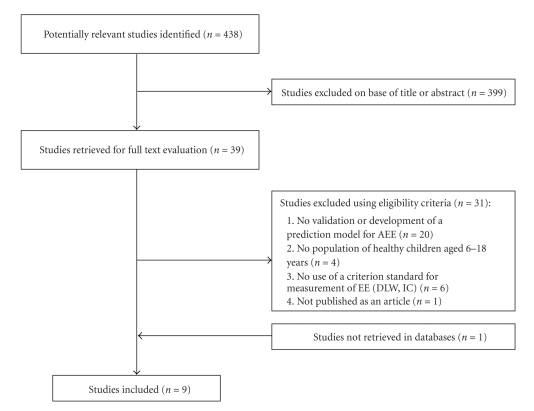
Selection process for studies included in the review.

The literature search identified 438 studies, after judgement based on title and abstract 39 studies remained (see Figure 1). Twenty studies were excluded after reading the full text due to deviating main purposes. Four studies were excluded because the population did not consist of healthy children aged 6–18 year. Six studies were excluded because the predicted AEE was not compared to a criterion measure of AEE as doubly labelled water or indirect calorimetry. Finally, one study was not published as an article but as a dissertation and therefore excluded.

In total eight studies were selected as eligible [10, 11, 13–18]. One additional study was not retrieved in the databases, it was already in possession of the author [9]. Therefore this review included nine studies describing the validation and generalizability of prediction models for the assessment of AEE in children and adolescents (see Appendix B).

All included studies had a cross-sectional research design. In total twenty-eight different prediction models were described. Two studies assessed the generalizability of previously published prediction models [11, 14]. In eight studies new prediction models were derived [9, 10, 13–18]. Some authors performed additionally a cross-validation analysis to assess the reliability of the new retrieved prediction model [13–15, 18]. Two studies retrieved data free-living with doubly labelled water as a criterion measure [11, 14]. The remaining studies [9–11, 13, 14, 18] were set in a controlled laboratory environment and used portable indirect calorimetry equipment as criterion measure, Puyau et al. [10, 17] additionally used room respiration calorimetry.

The included studies described six different accelerometers; two omnidirectional (Actical, Actiwatch), two uniaxial (Actigraph/CSA, Caltrac) and one triaxial (RT3). For the Actiheart this property could not be retrieved from the included studies.

The score on the checklist regarding methodological issues ranged from 5.0 to 8.0 (median 5.5 ± 2.0 IR). None of the included studies reported the amount of missing/lost data due to (malfunctioning of) the motion sensor, or refusal rate or the compliance rate of wearing the motion sensor. Due to this no conclusions could be made regarding feasibility. To review the validity and generalizability of the prediction models, the models were ordered in a table by accelerometer (see Table 1).

### 3.2. Validity

Eleven prediction models regarding the Actical were derived in laboratory settings based on activities as handwriting, cleaning, playing a video game/Nintendo, and walking at different speeds and grades (treadmill and indoor track) [9, 10, 13]. *R* ^2^ ranged from 0.45 to 0.81 (mean 0.65), which indicates that the prediction models explained 45–81% of the variance in measured AEE. The model of Puyau et al. [10] explained the largest variance (81%) with a standard error of the estimate (SEE) of 0.0111 (kcal · kg^−1^ · min^−1^). This model was derived during activities as playing Nintendo, cleaning, treadmill walking, and running. The model included age, gender, and the counts of the Actical placed at the hip. Puyau et al. [10] concluded that this model provided a valid measurement of AEE on a group level, further development was needed to accurately predict AEE of individuals.

For the Actigraph/CSA accelerometer, seven models were derived. One model was based on free-living data compared to doubly labelled water [15]. Six models were based on data retrieved during activities as lying, sitting, Nintendo, arts and crafts, playing, and walking at different speeds and grades (treadmill) [13, 14, 17]. *R* ^2^ ranged from  .37–.87. The prediction model of Corder et al. [14] explained with 87% the largest variance in measured AEE with a root mean square error (RMSE) of 118.0 J · kg^−1^ · min^−1^. This model contained accelerometer counts of the Actigraph placed at the hip, and height (cm) of the child. This model was derived during various intensity activities like lying, sitting, slow and brisk walking, jogging and hopscotch. The study by Corder et al. [14] compared models based on accelerometer counts solely and models that combined accelerometer counts with heart rate. The authors concluded that the combined models may be more accurate and widely applicable than those based on accelerometers alone.

The model by Ekelund et al. [15], derived during free living activities (fourteen consecutive days) explained 45% of the variance in measured AEE. There was a mean difference of −45 kcal · d^−1^ with large limits of agreement; −485 to 395 kcal · d^−1^.

The studies of Corder et al. [13, 14] derived six prediction models for the Actiheart, one model (Actiheart Activity) did not contain heart rate, the remaining five combined heart rate and activity counts. *R* ^2^ ranged from 0.69–0.91. The explained variance in measured AEE was the lowest for the Actiheart Activity model without heart rate (69%). The range of *R* ^2^ of the five models including heart rate was 0.86–0.91, thus an explained variance in measured AEE of 86–91%. The model with the largest explained variance (91%) consisted of heart rate, counts, and gender (RMSE 97.3 J · kg^−1^ · min^−1^). The derivation activities were lying, sitting, slow and brisk walking, jogging and hopscotch. Additionally a step test was performed for calibration. Despite systematic error, Corder et al. [14] concluded that these models can be used to predict overall AEE on a group level, during the six activities used in this protocol.

The studies of Puyau et al. [10, 17] derived three models for the Actiwatch. One study compared estimations from the Actiwatch placed at the hip and at the leg (fibula head) [17]. In the other study the Actiwatch was only placed at the hip [10]. The range of explained variance in measured AEE was 71%–81%. Highest explained variance was obtained when the Actiwatch was placed at the hip. A combination of both locations raised the explained variance of measured AEE to 84%. Puyau et al. [17] regarded this as a marginally improvement, not worth the increased cost, time, and effort. Puyau et al.[17] found the regression of AEE on counts to be independent of age and sex, therefore the prediction model was based on Actiwatch counts alone. Given the large standard of the estimate (SEE 0.0147 kcal · kg^−1^ · min^−1^) the prediction model was inappropriate for individuals.

The study of Johnson et al. [16] aimed to derive a prediction model for the Caltrac during free-living. Since the Caltrac counts showed no significant correlation with measured AEE, a prediction model without counts was derived. This model explained 28% of the variance in measured AEE by doubly labelled water, and consisted of gender, race/ethnicity (Caucasian, Mohawk), fat mass and fat free mass. When the mean Caltrac counts were forced in the model the explained variation in measured AEE still was only 29% making it unacceptable as an estimate of AEE.

Two prediction models were derived by the study of Sun et al. [18] for the RT3 accelerometer, one concerning indoor activities, one concerning outdoor activities. The indoor model explained 90% of the variance in measured AEE, the outdoor model 61%. Both models contained counts and body weight. Sun et al. concluded that despite underestimation of AEE during sedentary activities and overestimation of AEE in moderate, and vigorous activities by the RT3, their results indicated that the RT3 accelerometer might be used to provide acceptable estimates of physical activity in children.

### 3.3. Generalizability

Corder et al. [14] analysed the generalizability of the prediction models from Puyau et al. [17] and Trost et al. [19]. Puyau's model was originally derived with various sedentary, light, moderate and vigorous activities. In the protocol of Corder et al. the Puyau model explained 84% of the variance in measured AEE (RMSE 245.3 J · kg^−1^ · min^−1^). Mean bias was -151.6 J · kg^−1^ · min^−1^, the limits of agreements were −160.4 to −142.8 J · kg^−1^ · min^−1^. The Trost model (85%) was the most accurate of the two with the lowest RMSE (126.0 J · kg^−1^ · min^−1^). Mean bias on ratio scale was 5.5, the limits of agreement were −3.6 to 14.6. In the study of Corder et al. [14] most of the accelerometer counts models overestimated AEE during sedentary activities, and all the accelerometer counts models underestimated AEE for high-intensity activities, to the greatest extent during jogging. This was a systematic error, an intensity or activity dependent error. This bias and large range of the 95% ratio limits of agreement suggested that the models are only accurate for the assessment of group-level AEE.

Trost et al. [11] analysed the same Puyau et al. [17] model for the ActiGraph/CSA hip in their study concerning over ground walking and running. An overall mean pure error of 0.049 kcal · kg^−1^ · min^−1^ was found. (The pure error is calculated as the square root of the sum of squared differences between the observed and predicted values divided by the number of observations. The smaller the pure error, the greater the accuracy of the equation when applied to an independent sample [11].)

Mean bias on ratio scale was 1.33 (a difference between measured and predicted AEE of +33%). The corresponding limits of agreement were 0.44–2.22. Thus for any individual in the population, AEE values predicted by the Puyau et al. [17] model may differ from measured AEE values by −56 to +122%. Based on these findings Trost et al. [11] concluded that this prediction model does not accurately predict AEE during over ground walking and running. The model might be useful however for estimating participating in moderate and vigorous activity.

## 4. Discussion

This review shows that accelerometer-based prediction models can explain up to 91% [14] of the variance in measured AEE in children. Models derived in laboratory settings, using structured activities, provide estimations of AEE up to 91% [14], models derived free-living provide estimations of AEE up to 45% [15]. Laboratory-based models that explained ≥90% of the variance in measured AEE included heart rate [13, 14] or were based on the counts of a triaxial accelerometer (RT3) combined with body weight [18].

The difference found between laboratory-based models and free-living models might be explained by the derivation activities and the limitations of accelerometers. AEE predicted by a linear model, is likely to be more accurate when this model is derived and applied on a limited set of structured activities such as running and walking [20]. Activities free-living are much more complex and various then those included in a laboratory protocol. Moreover, the known limitations of accelerometers might cause deviations in the estimation of AEE free-living. Most accelerometers are mainly sensitive for accelerations in the vertical plane and less sensitive for more complex movements [13]. Accelerometers are limited in sensing activities as walking or cycling on a gradient. Also an increase in EE without a proportional increase in the amount of body movement is not detected (load-carrying, pushing and lifting objects) which causes estimation errors [13, 17]. 

Our findings suggest that the accuracy of the prediction model seems improved when a triaxial accelerometer is used. A triaxial accelerometer captures more movements than uniaxial and omnidirectional accelerometers. In the review of Westerterp [21] was concluded that the triaxial accelerometer can distinguish differences in activity levels in individuals. Especially sedentary activities were better reflected with a triaxial accelerometer than with an uniaxial accelerometer.

Models that included heart rate explained 86–90% of the variance in measured AEE [13, 14]. Due to the limitations of accelerometers there is no linear relation between the accelerometer counts and the measured AEE, adding heart rate in the prediction model may provide more accuracy [13, 14]. Corder et al. found a systematic error in all used prediction models which was intensity dependent. This systematic error was larger for the models without heart rate. The accelerometer counts models seemed more dependent on the activities tested (intensity), whereas the combined models (counts en heart rate) seemed more dependent on participant characteristics. The combined models may be more accurate and widely applicable [14].

The generalizability of the models is however limited and seems mainly dependent on the derivation activities. Nilsson et al. [20] compared several accelerometry prediction models in a large sample of children (*n* = 1321) during free-living in four different countries. The predicted AEE differ substantially between the models.

Free-living studies are most likely to represent actual daily activities performed by children. The laboratory-placed studies, included in this review, attempted to represent these activities by including locomotion activities [9–11, 13, 14, 17, 18], sports activities [10, 14, 17, 18], and recreational activities like playing video or computer games [9;10;17]. It remains however debatable whether treadmill walking [9, 10, 13, 17, 18] and cleaning activities [9, 10] actually represent the physical activity and the resulting AEE of activities daily performed by children. The chosen derivation activities will affect the linear relation between the accelerometer counts and AEE [20]. Nilsson et al. state that it is therefore unlikely that laboratory-based prediction models, using specific activities, are valid throughout the range of free-living activities [20].

Free-living studies estimate AEE by subtracting REE from total energy expenditure (TEE) provided by the doubly labelled water method [15, 16]. The included laboratory placed studies estimated AEE by subtracting REE from the EE provided by indirect calorimetry [9–11, 13, 14, 17, 18]. Seven of the included studies measured REE [9, 10, 13, 14, 16–18]. In two studies REE was predicted by the Schofield prediction equations [11, 15, 22]. The Schofield equations have good agreement with measured REE in healthy children and adolescents [23]. However, when indirect calorimetry is available, measurement of REE is preferred and more accurate, especially in children with chronic disease and movement disorders [24–26]. 

Measurement is more accurate since REE can vary with age, maturation, body mass, and level of physical activity [9]. Obviously a better estimation of AEE is obtained with a more accurate, measured REE.

Implication for clinicians is that previously published prediction models have limited applicability. Laboratory-based models can be used, on a group level, to predict AEE during specific activities, similar to the derivation activities. The use of a model combining accelerometer counts and heart rate, or a model combining triaxial accelerometer counts with body weight enhances validity. Generalizability of the models during free-living, however, is very limited. This is a significant limitation because measurement during free living is important to examine the dose-response relationship between physical activity and health-related fitness. The model derived by Ekelund et al. can be used, on a group level, for the prediction of AEE during free-living in 9-year-old children [15]. As stated before this model explained 45% of the variance in measured AEE.

Future development of prediction models applicable to free-living scenarios is needed. Future free-living studies should concern prediction models combining accelerometer counts and heart rate, or the counts of a triaxial accelerometer. As stated by Corder et al. especially the combination of accelerometer counts and heart rate might provide a more accurate and widely applicable model [14].

Regarding the reporting of findings, future recommendation is the description of the correlation between counts and measured AEE, since the counts are part of the prediction model. The limitations of the accelerometer itself may cause less accuracy, and therefore a less accurate prediction of AEE by the model.

To assess feasibility, authors should also report the amount missing and lost data due to malfunctioning of the motion sensor. Regarding free-living studies is additionally the refusal rate, or compliance rate with wearing the motion sensor interesting for clinicians.

## 5. Conclusion

Accelerometry based prediction models may provide an accurate estimate of AEE in children on a group level. The estimation of AEE is more accurate when the model is derived (and used) in a laboratory setting. The best results are retrieved when the model combines accelerometer counts with heart rate or when a triaxial accelerometer is used. Generalizability of the models during free-living however is limited. Future development of equations applicable during free-living is needed.

There are no professional relationships with companies or manufacturers who will benefit from the results of the present study.

## Figures and Tables

**Table 1:** Prediction models ordered by accelerometer.

Accelerometer	Activities	Criterion	Prediction models & Statistics
*Actical* (Mini Mitter Co., Inc., Bend, OR), (formerly known as Actiwatch). Omnidirectional: senses motions in all directions but is most sensitive within a single plane. Detects low frequency (0.5–3.2 Hz) G-forces (0.05–2.0 Hz) common to human movement and generates an analogue voltage signal, that is, filtered and amplified before being digitized by an A-to-D converter at 32 Hz. The digitized values are then summed over user-specified time intervals (epoch) between 0.25 and 1 min. The actual numbers stored by the Actical are proportional to the magnitude and duration of the sensed accelerations and, thus, roughly correspond to changes in physical activity energy expenditure. When mounted to the hip, most sensitive to vertical movements of the torso. Water resistant, lightweight (17 g), small (2.8 × 2.7 × 1.0 cm^3^).	Flat walking, graded walking, and running on a treadmill.	Indirect calorimetry	Corder et al. [13] AEE (J · kg − 1 · min^−1^) = 0.2AC + 168.7 →*R* ^2^ = 0.67, SEE 105 Flat walking: mean difference (J · kg^−1^ · min^−1^) −65 ± 23, 95% CI −78, −52 Graded walking: mean difference (J · kg^−1^ · min^−1^) 72 ± 35, 95% CI 52, 91 Running: Mean difference (J · kg^−1^ · min^−1^) 18 ± 69, 95% CI −26, 62 Flat and graded walking significantly different from measured values.
	- Three sitting activities: handwriting, card sorting, and Video game playing. - Three simulated house cleaning activities: floor sweeping, carpet vacuuming, table dusting. and - Locomotion activities: slow and moderate treadmill walking, treadmill jogging, OR self-paced slow walking, self-paced fast walking (indoor track).	Indirect calorimetry	Heil et al. [9], 1*R* = single regression modelling, 2*R* = double regression modelling Include sitting and cleaning activities Ankle 2*R*: AEE (kcal · kg^−1^ · min^−1^) = 0.02304 + (3.750E-5) × AC →*R* ^2^ =.60, SEE = 0.020, *P* < .001 Hip 2*R*: AEE (kcal · kg^−1^ · min^−1^) = 0.01667 + (5.103E-5) × AC →*R* ^2^ =.75, SEE = 0.014, *P* < .001 Wrist 2*R*: AEE (kcal · kg^−1^ · min^−1^) = 0.01149 + (3.236E-5) × AC →*R* ^2^ =.59, SEE = 0.020, *P* < .001 Include all activities Ankle 1*R*: AEE (kcal · kg^−1^ · min^−1^) = 0.03403 + (1.179E-5) × AC →*R* ^2^ =.45, SEE=0.028, *P* < .001 Hip 1*R*: AEE (kcal · kg^−1^ · min^−1^) = 0.03411 + (1.270E-5) × AC →*R* ^2^ =.61, SEE=0.024, *P* < .001 Wrist 1*R*: AEE (kcal · kg^−1^ · min^−1^) = 0.02299 + (1.902E-5) × AC →*R* ^2^ =.67, SEE=0.022, *P* < .001 Include walking and jogging activities Hip 2*R*: AEE (kcal · kg^−1^ · min^−1^) = 0.03534 + (1.135E-5) × AC →*R* ^2^, SEE = 0.018, *P* < .001 Include walking activities only Ankle 2*R*: AEE (kcal · kg^−1^ · min^−1^) = −0.02268 + (1.939E-5) × AC→*R* ^2^ =.60, SEE = 0.015, *P* < .001 Wrist 2*R*: AEE (kcal · kg^−1^ · min^−1^) = 0.03115 + (1.581E-5) × AC →*R* ^2^ =.69, SEE = 0.019, *P* < .001
	Playing Nintendo, using a computer, cleaning, aerobic exercise, ball toss, treadmill walking, and running.	Room respiration calorimetry 4 h, Indirect calorimetry 1 h.	Puyau et al. [10] Hip: AEE (kcal · kg^−1^ · min^−1^)=0.00423+0.00031*Actical^0,653^→*R* ^2^ 0.811, SEE 0.0110 Counts, age, and gender were included in the model; inclusion of height gave no significant improvement.
*ActiGraph* (model 7164, formerly known as Computer Science and Applications *CSA* activity monitor. Manufacturing Technologies Inc. health Systems, Shalimar, FL) Uniaxial. Is sensitive to movements in the 0.51–3.6 Hz range. Hip- and ankle-mounted. When mounted to the hip, most sensitive to vertical movements of the torso. The acceleration signal is represented by an analog voltage that is sampled and digitized by an eight-bit analog-to-digital converter at a rate of 10 times per second.	Flat walking, graded walking, and running on a treadmill.	Indirect calorimetry	Corder et al. [13] Hip: AEE (J · kg^−1^ · min^−1^) = 0.17 counts + 201.1 →*R* ^2^ = 0.5, SEE 123 Flat walking: mean difference (J · kg^−1^ · min^−1^) −88 ± 14, 95% CI −97, 80 Graded walking: mean difference (J · kg^−1^ · min^−1^) 66 ± 26, 95% CI 51,82 Running: Mean difference (J · kg^−1^ · min^−1^) 139 ± 60, 95% CI 101, 177 All significantly different from measured values. Ankle: AEE (J · kg^−1^ · min^−1^) = 0.89 counts + 39.4 gender – 1.4 height + 361.9 →*R* ^2^ = 0.37, SEE 144 Flat walking: mean difference (J · kg^−1^ · min^−1^) −104 ± 39, 95% CI −126, −82 Graded walking: mean difference (J · kg^−1^ · min^−1^) 77 ± 48, 95% CI 51, 103 Running: Mean difference (J · kg^−1^ · min^−1^) 176 ± 233, 95% CI 28, 324 All significantly different from measured values. Age was not included in the models because of lack of heterogeneity in the sample.
	Six activities, each activity lasted 5 minutes: Lying, sitting, slow walking, brisk walking, jogging, hopscotch. (Step test calibration 8 minutes).	Indirect calorimetry	Corder et al. [14] validation of priori models and one new model derived. Corder et al. [13], hip: AEE (J · kg^−1^ · min^−1^) = 0.054 × AC[counts per minute] + 169 →*R* ^2^ = 0.81, RMSE = 161.8 Mean bias: −44.8; 95% CI: −54.1, −35.5 Derivation activities: flat and graded treadmill walking and flat running Puyau et al. [17], hip: AEE (J · kg^−1^ · min^−1^) = 0.042 × AC[counts per minute] + 76.6 →*R* ^2^ = 0.84, RMSE = 245.3 Mean bias: −151.6; 95% CI: −160.4, −142.8 Derivation activities: various sedentary, light, moderate, and vigorous activities Trost et al. [19], hip: AEE (J · kg^−1^ · min^−1^) = 3.35 × AC[counts per minute] + 334.8 × weight [kg] − 9334 →*R* ^2^ = 0.85, RMSE = 126.0 Mean bias: 5.5; 95% CI: −3.6, 14.6 Derivation activities: flat treadmill activity at 3.2, 6.4, and 9.6 km^−1^ Corder et al. [14], hip: AEE (J · kg^−1^ · min^−1^) = 0.1 × AC[counts per minute] − 2.29 × height [cm] + 353 →*R* ^2^ = 0.87, RMSE 118.0 Mean bias −1,9; 95% CI −11.4, 7,6 Derivation activities: lying, sitting, slow and brisk walking, jogging and hopscotch
	Free-living; Two school weeks, 14 consecutive days, the children wore the monitor during daytime following their normal living. Exceptions were during water activities such as swimming and bathing.	Doubly labelled water	Ekelund et al. [15] Centre of gravity/lower back: AEE (kcal · d^−1^) = (Activity counts × 1.042) − (Gender × 243.4) + 238 → Adjusted *R* ^2^ = 0.45, SEE 149 The mean difference between measured and predicted AEE was −45 kcal · d^−1^ (*P* = .58), and the 95% limits of agreement were −485 kcal · d^−1^ to 395 kcal · d^−1^.
	Sedentary: Nintendo, arts and crafts, playtime 1 Light activities: aerobic warm-up, walk 1 Moderate activities: Tae Bo exercises, playtime 2, walk 2 Vigorous activity: jogging.	Room respiration calorimetry	Puyau et al. [17] Hip: AEE (kcal/kg/min) = 0.0183 + 0.000010 (counts) → SEE 0.0172 →*r* ^2^ (adj) 75% Fibula head: AEE (kcal/kg/min) = 0.0142 + 0.000007 (counts) → SEE 0.0154 → *r* ^2^ (adj) 82% Predicting AEE from the combination of the counts from the hip and leg increased the *r* ^2^ (adj) to 86%. Regression of AEE on counts was independent of gender and age, thus only counts were included in the model.
	Field conditions; flat oval indoor track. Normal walking, brisk walking, easy running, fast running. The intensity of each task was self-selected.	Indirect calorimetry	Trost et al. [11] validation of Puyau et al. 2002 Puyau et al. [17], hip: AEE (kcal · kg^−1^ · min^−1^) = 0.0183 + 0.000010 (counts per minute)*t*-tests for difference in means of measured AEE (indirect calorimetry) and predicted AEE by Puyau equation: Normal walking: 0.6% not significantly different (pure error 0.014 kcal · kg^−1^ · min^−1^) Brisk walking −13.3% significantly different (pure error 0.025 kcal · kg^−1^ · min^−1^) Slow running −29.3% significantly different (pure error 0.054 kcal · kg^−1^ · min^−1^) Fast running −37.7% significantly different (pure error 0.078 kcal · kg^−1^ · min^−1^) Overall mean pure error was 0.049 kcal · kg^−1^ · min^−1^ Mean bias on ratio scale is 1.33, difference between measured and predicted AEE was +33%. The corresponding 95% ratio limits of agreement were 0.44–2.22
*Actiheart* (Cambridge Neurotechnology, Cambridge, UK). Combined HR and movement sensor is able to measure acceleration, HR, HR variability, and ECG magnitude. Acceleration is measured by a piezoelectric element with a frequency range of 1–7 Hz (3 dB). One electrode is placed at the base of the child's sternum and the other horizontally to the child's left side. The main component is 7 mm thick with a diameter of 33 mm. A wire of approximately 100 mm length runs to the clip (5 × 11 × 22 mm^3^). The total weight is 8 g.	Flat walking, graded walking, and running on a treadmill (protocol).	Indirect calorimetry	Corder et al. [13] Actiheart Activity, chest: AEE (J · kg^−1^ · min^−1^) = 0.22 counts + 29.3 gender + 144.3 →*R* ^2^ = 0.69, SEE 101 Flat walking: mean difference (J · kg^−1^ · min^−1^) −74 ± 32, 95% CI −91,−56 Graded walking: mean difference (J · kg^−1^ · min^−1^) 56 ± 32, 95% CI 38, 74 Running: Mean difference (J · kg^−1^ · min^−1^) −86 ± 116, 95% CI −159, −12 All significantly different from measured values. Actiheart Combined, chest: AEE (J · kg^−1^ · min^−1^) = 4.4 HRAR + 0.08 counts − 2.7 gender + 1.1 (gender × HRAR) + 15.1 (HRAR: Heart Rate Above Rest) →*R* ^2^ = 0.86, SEE 69 (69 J · kg^−1^ · min^−1^) Flat walking: mean difference (J · kg^−1^ · min^−1^) −11 ± 27, 95% CI −55, −15 Graded walking: mean difference (J · kg^−1^ · min^−1^) −38 ± 48, 95% CI −29, 26 Running: mean difference (J · kg^−1^ · min^−1^) 10 ± 102, 95% CI −105, −6 Graded walking significantly different from measured values. (Age was not included in the models because of lack of heterogeneity in the sample).
	Six activities, each activity lasted 5 minutes: Lying, sitting, slow walking, brisk walking, jogging, hopscotch. (Step test calibration 8 minutes).	Indirect calorimetry	Corder et al. [14] validation of priori models and new models derived. Corder et al. [13], chest: HR + ACC model: AEE (J · kg^−1^ · min^−1^) = 5.6 × HRaS [bpm] + 1.37 × gender*HRaS + 0.1 × AC[counts per minute] − 44 × gender − 129 (HRaS: Heart Rate above Sleep) →*R* ^2^ = 0.90 RMSE = 118.0 Mean bias: 18.7; 95% CI: 8.1, 29.3 Derivation activities: flat and graded treadmill walking and flat running Corder et al.[13]/Branched, chest: HR equation: AEE (J · kg^−1^ · min^−1^) = 6.2 × HRaS [bpm] – 27 × gender + 1.2 × gender*HRaS − 139 AC equation: AEE (J · kg^−1^ · min^−1^) = 0.22 × AC[counts per minute] + 29 × gender + 144 *R* ^2^ = 0.90, RMSE = 115.6 Mean bias: −43,4; 95% CI: −52.2, −34.6 Derivation activities: flat and graded treadmill walking and flat running (First branch threshold is 25 activity counts per minute, the second depends on the HRaS) Corder et al. [14], chest: AEE (J · kg^−1^ · min^−1^) = 5.17x HRaS [bpm] + 0.61 × gender*HRaS + 0.07 × AC[counts per minute] −0.6 × gender − 74 →*R* ^2^ = 0.90, RMSE = 100.1 Mean bias −2.5; 95% CI: −12.2, 7.2 Derivation activities: lying, sitting, slow and brisk walking, jogging and hopscotch Corder et al. [14], chest: AEE (J · kg^−1^ · min^−1^) = 3.95 × HRaS[bpm] + 0.26 × gender*HRaS + 0.07 × AC[counts per minute] + 8 × gender + 0.68 ×*α*-step + 1.31 × *β*-step* HRaS −49 *R* ^2^ = 0.91, RMSE = 97.3 Mean bias: −2.3; 95%CI: −11.4, 6.8 Derivation activities: Lying, sitting, slow and brisk walking, jogging and hopscotch (with step calibration)
*Actiwatch* (model AW16; Mini-Mitter, Bend Or). Omnidirectional accelerometer built from a cantilevered rectangular piezoelectric bimorph plate and seismic mass, which is sensitive to movement in all directions, but most sensitive in the direction parallel with the longest dimension of the case. Is designed to detect a wide range of limb movements related to sleep/wake behavior. Sensitive to movements in the 0.5- to 7-Hz frequency range. Firmware detects the peak value of 32 samples in a 1-s window and adds this to the accumulated value for that epoch. Waterproof.	Sedentary: Nintendo, arts and crafts, playtime 1 Light activities: aerobic warm-up, walk 1 Moderate activities: Tae Bo exercises, playtime 2, walk 2 Vigorous activity: Jogging.	Room respiration calorimetry	Puyau et al. [17] Hip: AEE (J · kg^−1^ · min^−1^) = 0.0144 + 0.000038 hip (counts) → SEE 0.0147 →*r* ^2^(adj) 81% Fibula head: AEE (J · kg^−1^ · min^−1^) = 0.0143 + 0.000020 leg (counts) → SEE 0.0195 →*r* ^2^(adj) 71% Predicting AEE from the combination of the counts from the hip and leg increased the *r* ^2^(adj) to 84%. Regression of AEE on counts was independent of gender and age, thus only counts were included in the model.
	Playing Nintendo, using a computer, cleaning, aerobic exercise, ball toss, treadmill walking and running.	Room respiration calorimetry 4 h, Indirect calorimetry 1 h.	Puyau [10] Hip: AEE (kcal · kg^−1^ · min^−1^) = 0.00441 + 0.00032*Actiwatch^0,724^→*r* ^2^ 0.79, SEE 0.0117 Counts, age and height were included in the model, inclusion of gender gave no significant improvement.
*Caltrac* accelerometer (Muscle Dynamics Fitness, Madison, Wis, USA). Measures the degree and intensity of movement in the vertical plane.	Free-living; Three days, including one weekend day. The subjects began wearing the Caltrac upon waking in the morning and continued until just before going to sleep at night. The Caltrac was taken off for activities involving water, such as swimming or bathing.	Doubly labelled water	Johnson et al. [16] Hip: AEE (kcal · d) = 63.97+ (284.962 × gender) − (17.671 × race) + (12.876 × FM) − (6.18 × FFM) →*R* ^2^ = 0.28, *P* = .06, SSE ± 315 Race: Caucasian = 0, Mohawk = 1 When the three-day mean AC was forced into the model, the amount of variation in AEE was explained, did not increase significantly *R* ^2^ = 0.29, *P* = .12, SSE = ±321
*RT3* accelerometer (Stayhealthy, Monrovia, CA). The instrument measures the acceleration in three dimensions: anterior-posterior (x), medio-lateral (y), and vertical (z) directions. Activity counts is the square root of the sum of the squared accelerations of each direction.	Indoor: laying down, sitting relaxed, writing, standing relaxed, sitting and standing (alternating every 5 s), cycling, stepping up and down, walking. The speed of treadmill was predetermined so that most children could complete jogging on the treadmill. Outdoor: picking up tennis balls, and then standing up, catching and passing a basketball, kicking a soccer ball, shooting a basketball while walking, walking relaxed nonlinearity, jogging lightly, and jogging fast.	Indirect calorimetry	Sun et al. [18] The RT3 was placed at the waist/midline thigh. Indoor activities: AEE (kcal · min^−1^) = 0.0006359 (counts · min^−1^) −0.0006427 (body weight) + 0.733 (Activity counts is the square root of the sum of the squared accelerations of each direction) *r*=.95 (*R* ^2^ =.90), *P* = .001. B&A: mean error 0.94 kcal · min^−1^, 95% confidence interval = (−1.83,2.77) kcal · min^−1^. Outdoor activities: AEE (kcal · min^−1^) = 0.00030397 (counts · min^−1^) + 0.00586272 (body weight) + 0.58 *r*=.78 (*R* ^2^ =.61), *P* < .001. B&A: mean error −1.66 kcal · min^−1^, 95% confidence interval (−3.2, 1.57) kcal · min^−1^.

Abbreviations: AC: Accelerometer Counts, adj.: adjusted, AEE: Activity related Energy Expenditure, B&A: Bland & Altman, bpm: beats per minute, CI: Confidence Interval, FFM: Fat Free Mass, FM: Fat Mass, g: gram, h: hour, Hz: hertz, J: Joule, kcal: Kilocalorie, kg: kilogram, min: minute, r= correlation coefficient, RMSE: Root Mean Squared Error, SEE: Standard Error of the Estimate, SSE: Sum of Squared Errors.

**Table 2:** Items concerning study design.

*1*		*Sample characteristics (* *n* *, sex, age, weight, height, BMI% body fat/sum of skin folds, health status)*

	1	≥6 sample characteristics are described (at least: *n*, sex, age, weight, and height)
	0.5	4-5 sample characteristics are described
	0	≤3 sample characteristics are described

*2*		*Protocol*

	1	Information on setting, activities, duration (days or hours), and period of wearing the motion sensor
	0.5	Information on period of wearing the motion sensor is missing
	0	Not clear at all

*3*		*Measurements*

	1	Complete information on motion sensor (type, output, epoch, placement) and reference method(s) (type, output)
	0.5	Some information on motions sensor (type, output, epoch, placement) and reference method(s) (type, output) is missing
	0	Very limited information on motion sensor (type, output, epoch, placement) and reference method(s) (type, output)

*4*		*Statistical analyses*

	1	Complete information on statistical analysis (tests, subgroup analysis), statistical software package and *P*-value
	0.5	Some information on statistical analyses (tests, subgroup analysis), statistical software package and *P*-value
	0	Very limited information on statistical analysis (tests, subgroup analysis), statistical software package and *P*-value

**Table 3:** Items concerning validity.

*5*		*Is “criterion” validity reported for the prediction model?*

	1	Yes
	0	No

*6*		*Adequate measure of validity?*

	1	Sensitivity
	1	Specificity
	1	Pearson's product-moment correlation coefficient
	1	Spearman's rank order correlation coefficient
	0.5	95% limits of agreement (Bland and Altman)
	0	Other measure

*7*		*Acceptable level of criterion validity?*

	+	*r* ≥ 0.60
	±	*r* = 0.30–0.60
	−	*r* < 0.30
*8*		*Is reliability reported for the prediction model? (cross validation analysis)*
	1	Yes
	0	No

*9*		*Adequate measure for reliability?*

	1	Intraclass correlation coefficients
	1	95% limits of agreement (Bland and Altman)
	1	Cohen's Kappa
	1	Standard error of measurement
	1	Coefficient of variation
	0	Pearson's product-moment correlation coefficient
	0	Spearman's rank order correlation coefficient
	0	Kendall's tau
	0	Other measure

*10*		*Acceptable level of reliability?*

	+	ICC ≥ 0.70
	±	ICC = 0.40–0.70
	−	ICC < 0.40

**Table 4:** Items concerning feasibility.

*11*		*Is the amount of missing/lost data due to (malfunctioning of) the motion sensor reported/reducible?*

	1	Yes
	0	No

*12*		*Acceptable amount of missing/ lost data?*

	+	≤5%
	−	>5%

*13*		*Is the refusal rate of, or the compliance rate with wearing the motion sensor reported?*

	1	Yes
	0	No

*14*		*Acceptable refusal rate?*

	+	<15%
	±	15–30%
	−	≥30%

**Table 5:** Data of included studies.

Study	Population	Score checklist (out of 10)	Setting	Accelerometer (placement)	Prediction model(s)	Conclusion authors
Corder et al. [13]	39 children aged 13.2 ± 0.3 yr, 23♂, 16♀.	7.5	Laboratory setting.	*Actiheart* (chest) *Actigraph* (hip, ankle) *Actical* (hip)	Six prediction models were derived, one not consisting of accelerometer counts, this one was excluded.	Corder et al. concluded that the combined HR and activity monitor Actiheart is valid for estimating AEE in children during treadmill walking and running. The combination of HR and activity counts provides the most accurate estimate of AEE as compared with accelerometry measures alone.
Corder et al. [14]	145 children aged 12.4 ± 0.2 yr, 66♂, 79♀.	7.5	Laboratory setting.	*Actigraph* (hip) *Actiheart* (chest)	Five previously published prediction models (Coder et al. [13](3), Puyau et al. [17], Trost et al. 19)Three derived using the current data.	Corder et al. concluded that the ACC and HR + ACC can both be used to predict overall AEE during these six activities in children; however, systematic error was present in all predictions. Although both ACC and HR + ACC provides accurate predictions of overall AEE, according to the activities in their study, Corder et al. concluded that AEE-prediction models using HR + ACC may be more accurate and widely applicable than those based on accelerometry alone.
Ekelund et al. [15]	26 children aged 9.1 ± 0,3 yr, 15♂, 11♀.	8.0	Free-living.	*Actigraph/CSA* (centre of gravity/lower back)	One prediction model derived.	Ekelund et al. concluded that activity counts contributed significantly to the explained variation in TEE and was the best predictor of AEE. Their cross-validation study showed no significant differences between predicted and measured AEE. However the relatively large SEE together with the wide limits of agreement preclude individual comparison. Ekelund et al. suggested therefore that the prediction equation could be used to assess the mean AEE on a group level.
Heil et al. [9]	24 children: 14♂ aged 12 ± 3 yr, 10♀ aged 13 ± 2 yr.	5.5	Laboratory setting.	*Actical* (wrist, ankle, hip)	Nine prediction models derived.	Heil et al. concluded that the proposed algorithms for the Actical appeared to predict AEE accurately whether worn at the ankle, hip or wrist. Additionally they state that their results however, are clearly limited by the laboratory nature of the data collection and need to be validated under free-living conditions. In practice, according to Heil et al., may provide their algorithms useful predictions of AEE for groups of children, but the tracking of individuals may still involve considerable error.
Johnson et al. [16]	31 children aged 8.3 ± 2.0 yr, 17♂, 14♀.	5.0	Free-living.	*Caltrac* (hip)	Sallis et al. 1989 equation; originally validated against HR, thus excluded in this study. One prediction model derived.	According to Johnson et al. their study failed to find a significant correlation between either activity counts and AEE or Caltrac average calories with AEE. Their major finding was that the Caltrac accelerometer was not a useful predictor of AEE in the sample. Johnson et al. concluded that the equation consistently overestimated AEE and had wide limits of agreement, making it unacceptable as an estimate of energy expended in physical activity for this sample.
Puyau et al. [17]	26 children 14♂ aged 10.7 ± 2.9 yr, 12♀ aged 11.1 ± 2.9 yr.	5.5	Laboratory setting.	*Actigraph/CSA * *Actiwatch* (both: hip, fibula head)	Four prediction models were derived.	Puyau et al. concluded that the high correlations between the activity counts and AEE demonstrates that the CSA and Actiwatch monitors strongly reflected energy expended in activity. Given the large SEE of the regression of AEE on activity counts, they found the prediction of AEE from CSA of Actiwatch activity counts inappropriate for individuals.
Puyau et al. [10]	32 children aged 7–18 yr, 14♂, 18♀.	5.5	Laboratory setting.	*Actiwatch Actical* (both: hip)	Two models derived.	Puyau et al. concluded that activity counts accounted for the majority of the variability in AEE with small contributions of age, sex, weight, and height. Overall, Actiwatch equations accounted for 79% and Actical equations for 81% of the variability in AEE. Relatively wide 95% prediction intervals for AEE showed considerable variability around the mean for the individual observations. Puyau et al. suggest that accelerometers are best applied to groups rather than individuals. According to Puyau et al. provided both accelerometer-based activity monitors valid measures of children's AEE but require further development to accurately predict AEE of individuals.
Sun et al. [18]	27 children aged 12–14 yr, 21♂, 6♀ (25 indoor, 18 outdoor).	8.0	Laboratory setting.	*RT3* (waist/midline thigh)	Two models derived and manufacturer's model was used. Since the manufacturer's model is not revealed it was excluded.	Sun et al. concluded that the results of their study show that the RT3 accelerometer provides a valid method to examine physical activity patterns qualitatively and quantitatively for children. The moderate to high correlation coefficients between the physical activities in various lifestyle conditions from this device and the metabolic costs in simulated free-living conditions strongly supports, according to Sun et al., that the RT3 accelerometer serves as a valid, objective measure of physical activity of children, even in a tropical environment such as Singapore.
Trost et al. [11]	45 children aged 13.7 ± 2,6 yr, 22♂, 23♀.	5.5	Laboratory setting.	*ActiGraph* (hip)	Validation of three models. Two models not concerning AEE were excluded. The model by Puyau et al. [17] was included.	Trost et al. concluded that previously published ActiGraph equations developed specifically for children and adolescents do not accurately predict AEE on a minute-by-minute basis during overground walking and running.

Abbreviations; ACC: Accelerometer, AEE: Activity related Energy Expenditure, HR: Heart Rate, SEE: Standard Error of the Estimate, TEE: Total Energy Expenditure, yr: year.

## Appendices

### A. Checklist Methodological Issues

Evaluation checklist for studies on prediction models for accelerometers

Source; Clinimetric review of motion sensors in children and adolescents. Journal of Clinical Epidemiology 59 (2006) 670–680. De Vries SI, Bakker I, Hopman-Rock M, Hirasing RA, van Mechelen W.

Modified by S. de Graauw, 2009.

See Tables 2–4.

### B. Data of Included Studies

See Table 5.
